# Crossover Analysis of the Astringent, Antimicrobial, and Anti-inflammatory Effects of Illicium verum/Star Anise in the Oral Cavity

**DOI:** 10.1155/2021/5510174

**Published:** 2021-05-30

**Authors:** Ali A. Assiry, Mohmed Isaqali Karobari, Shaeesta Khaleelahmed Bhavikatti, Anand Marya

**Affiliations:** ^1^Preventive Dental Science Department, Faculty of Dentistry, Najran University, Saudi Arabia; ^2^Conservative Dentistry Unit, School of Dental Sciences, Universiti Sains Malaysia, Health Campus, 16150 Kubang Kerian, Kelantan, Malaysia; ^3^Department of Periodontics, School of Dental Sciences, Universiti Sains Malaysia, Health Campus, 16150 Kubang Kerian, Kelantan, Malaysia; ^4^Department of Orthodontics, Faculty of Dentistry, University of Puthisastra, Phnom Penh, Cambodia; ^5^Department of Orthodontics, Saveetha Dental College, Saveetha Institute of Medical and Technical Sciences, Chennai, India

## Abstract

**Introduction:**

Illicium verum commonly known as star anise has been widely used in many Asian countries for pharmaceutical treatment for many diseases. The aim of the present study was to investigate the anti-inflammatory, astringent, and antimicrobial properties of an Illicium verum mouthwash.

**Methods:**

The present double blinded randomized clinical trial was conducted on fifty subjects, divided into groups A and B. Illicium verum mouthwash (group A) and placebo (group B) were provided to subjects for 21 days; after 14 days, washout period mouthwashes were switched as per crossover design between groups for 21 days. The gingival index (GI), papillary bleeding index (PBI), and oral microbial count were recorded at each stage of study.

**Results:**

The significant intragroup difference was observed, before crossover in group A and after crossover in group B for GI, PBI, and oral microbial count at different stages of study. On comparing both group A and group B at the first and second follow-up for GI, PBI, and oral microbial count, a statistically significant difference (*p* < 0.05) was observed. A statistically highly significant mean intergroup and intragroup difference was seen for all the clinical parameters at different stages of study.

**Conclusion:**

The study revealed that the Illicium verum/star anise has potent antibacterial, anti-inflammatory, and astringent properties.

## 1. Introduction

Ayurveda is the most ancient health care system in the world and is practiced widely in India, Sri Lanka, and other countries. There is a great demand for herbal medicines in developed as well developing countries because of their wide biological activities, safety margin than synthetic drugs, and lesser cost.

Illicium verum, also named as star anise, is the fruit of a medium-sized tree that was originally distributed across the tropical and subtropical areas of Asia. The genus was named Illicera (allure) probably because of its sweet and attractive fragrance [1]. Its extensive use for flavoring food has led to it being known exclusively as a culinary spice. Besides bearing a wide range of medicinal properties, its use is being contemplated across relatively newer areas of application. The use of herbal extracts as antimicrobial agents has distinct advantages: their natural origin and the associated low risk [2].

Star anise shows quite potent antibacterial properties [3]. Anethole, the main chemical constituent in star anise is believed to be the main antimicrobial agent. The link between oral diseases and the activities of microbial species that form part of the microbiota of the oral cavity is well established [4]. Dental disease prevention is commonly associated with a reduction of some Gram-positive and Gram-negative microorganisms. It is well documented that Illicium verum has an anti-inflammatory property [5]. Presently, no such study is available regarding the in vivo effect of star anise.

Thus, the aim of this study was to study the antimicrobial activity of star anise against oral microorganism and to test the efficacy of star anise as an astringent and anti-inflammatory agent.

## 2. Materials and Methods

### 2.1. Study Setting

The present research was a double blind randomized controlled trial conducted with an ethical approval number 20210049 from the Faculty of Dentistry, Najran University, to evaluate the antibacterial, anti-inflammatory, and astringent properties of an Illicium verum-based mouth rinse for a period of 70 days. Permission to conduct the study was obtained from the Ethical Review committee of the institute before the commencement of the trial.

### 2.2. Sample Size

Sample size was estimated considering the following variables, i.e., keeping coefficient of variation = 20%, confidence interval = 90%, level of significance = 0.05, and the power of the study = 80%. With this information, the minimum sample size required was 19 in each group. Therefore, after applying eligibility criteria and considering the unknown observer\instrumentation errors, the sample size was increased to 25 for each group.

### 2.3. Study Design

In the current study, first, all eligible subjects were being identified. Only those subjects were selected which fulfilled the inclusion and exclusion criteria. Sixty-two subjects were selected, out of which 12 failed to provide the positive consent. So, the final sample for the present study was fifty.

### 2.4. Inclusion Criteria

Subjects between the age group of 18 and 25 years, presenting no allergy, free from systemic diseases, those who were having moderate gingival scores, and fair plaque scores according to the plaque and gingival indices proposed by Silness and Loe 1964 and Loe and Silness 1963, respectively, and subjects formally agreed with all study aspects and signed the consent form were selected for the present study. Patients who had not undergone any oral hygiene sessions previously up to the last 6 months were included in the study.

### 2.5. Exclusion Criteria

Subjects who had been on medication in the last 6 months and who had history of antibiotic therapy in the previous 1 month till the start of the study were excluded.

### 2.6. Collection and Identification of Star Anise

Different samples of fresh-dried well-packed Illicium verum were purchased from the local market and departmental store. These samples were coded and sent for verification to the department of botany. The approved samples were further being used for the present study.

### 2.7. Preparation of Star Anise Extracts

Three hundred grams of the dried Illicium verum was milled to a course powder using an electrical grinder and was extracted with 6000 ml distilled waters. The adequate concentration of Illicium verum mouthwash was being identified by the antibacterial efficacy of the different concentration being tested. The solution was prepared at 1%, 3%, and 10% concentrations, and its efficacy was tested over Gram-positive and Gram-negative microorganisms. The most effective concentration (i.e., 3%) was being used for the present study.

The extract was filtered through Whatman filter paper No. 1. All residues were weighed, and the concentration of the final solution was 3% or 3 g/100 ml. A sweetening agent (2% sorbitol) and preservative (0.01% sodium methyl paraben) were added to obtain the final solution which was stored in an airtight container in the refrigerator at 4°C until used for further analyses.

### 2.8. Intervention

The selected participants were randomized into two groups (group A and group B). The subjects in group A were administered with a star anise-based mouthwash and subjects in group B allotted with placebo (color tinted water). Star anise mouthwash and placebo had identical appearance. Groups A and B were given star anise mouthwash and placebo by unaffiliated person, and its identity was obscured from investigator and participants, i.e., which group is receiving which mouthwash.

Selected subjects were instructed not to use any antimicrobial agent during at least 14 days before the study began. Subjects selected for the study were provided with a commercially available fluoridated dentifrice and a soft-bristled toothbrush for regular use for the duration of the study. Subjects were instructed to suspend the use of all other oral hygiene formulations during the study period.

After the 14 days washout phase, clinical scores for each parameter were obtained. Clinical parameters evaluated in the study were the antimicrobial property, anti-inflammatory property, and astringent property using colony-forming units (CFU) count, Loe and Silness Gingival Index [6], and papillary bleeding index [7], respectively.

To assess the antimicrobial property, the subjects were asked to spit their saliva into the sterile plastic containers. About 3–4 ml of whole unstimulated saliva was collected over 2 minutes and divided into samples. These samples were coded at the time of collection as well as processing. Once processed, these samples were analyzed in the pathology laboratory on the same day. Before the analysis was carried out, the samples were stored at normal room temperature, i.e., 17°C–25°C. For the analysis, each sample was sonicated (Vibra Cell 400 W, Sonics & Materials, Inc. –5% amplitude and 9.9 s cycles of 6 pulses each) followed by dilution in saline (100-1000 times) These diluted samples (5 *μ*l) were spread out on blood agar containing petri dishes. These dishes were incubated (Jovan IG 150) at 37°C for a period of 48 hours along with 10% CO^2^. After the 48-hour incubation period, the dishes were transferred to an aerobic incubator and maintained at 37°C for another 24-hour period. The number of microorganisms was evaluated using the dishes, and enumeration of the bacterial colonies and viable bacterial microorganisms was carried out.

**Figure 1:**
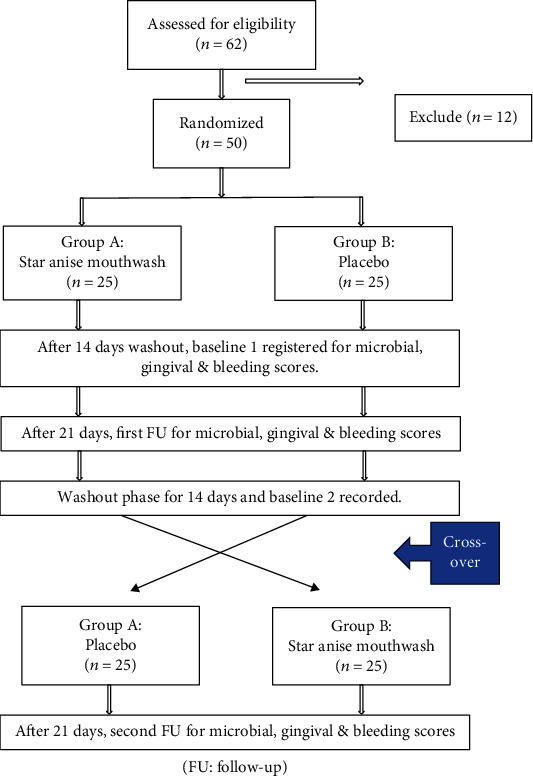
Flowchart showing procedural steps taken during clinical trial.

Subjects were given an adequate supply of their respective mouthwash and instructed to rinse with the mouthwashes for 30 seconds, in the morning and night (before going to bed) and not to eat or drink anything for at least half an hour after rinsing. Baseline data were recorded before commencement of intervention (baseline 1). After 21 days trial period, each subject returned for scoring of clinical parameters (first follow-up), followed by discontinuation of intervention, and instructed to continue routine oral hygiene aids. After 14 days washout period, again baseline data were assessed (baseline 2), and the mouthwashes (star anise mouthwash and placebo) were crossover in group A and group B. On second follow-up after 21 days, clinical parameters were recorded (Figure 1).

### 2.9. Statistical Analysis

Statistical analysis was done by using the mean and standard deviation (SD). Unpaired *t*-test was performed to determine the differences between different stages of study among various clinical parameters within a group and paired *t*-test for comparison of various clinical parameters at different stages between groups A and B. *p* value < 0.05 was considered statistically significant.

## 3. Result

In present study, the mean age of group A and group B was found to be 20.40 ± 1.44 and 20.56 ± 1.47 years, respectively. Male subjects were dominant in both the groups (Table 1).

The mean gingival index, papillary bleeding index, and microbial count between baseline 1 and first FU in group A were found to be 0.65 ± 0.05, 0.68 ± 0.05, and 0.64 ± 0.06 and 0.48 ± 0.04, 0.55 ± 0.03, and 0.47 ± 0.04, respectively. The statistically significant difference was observed (*p* < 0.05) in the gingival index, papillary bleeding index, and microbial count, when their change in score was assessed from baseline 1 to first follow-up in group A and baseline 2 to second follow-up in group B (Table 2).

Table 3 shows the intergroup comparison of the mean scores of various clinical parameters at different time intervals of study. A statistically significant mean difference was observed for all clinical parameters when they were compared at first FU and second FU between groups A and B, respectively.

When the mean intergroup difference from baseline 1 to first follow-up and baseline 2 to second follow-up was compared statistically for all clinical parameters between groups A and B, it was found to be statistically significant as *p* < 0.05. Likewise, highly significant intragroup mean findings were observed for groups A and B in relation to different clinical parameters (Table 4).

## 4. Discussion

Plaque is the main agent responsible for the breakdown of periodontal tissues leading to gingival and periodontal diseases. The removal of this plaque regularly is of paramount importance in the prevention of periodontal disease. The inability of the adult population to perform adequate mechanical tooth cleaning has stimulated the search for chemotherapeutic agents added to dentifrices to improve plaque control and prevent gingivitis. Herbal products are one group of agents which has been used extensively in reducing the bacterial population. Phytotherapeutic products have been investigated with these purposes and have shown satisfactory results [8]. This made us evaluate the efficacy of Illicium verum on periodontal health and its effect on oral microbial count.

So far, various studies have been performed on Illicium verum, and different properties were revealed such as antimicrobial, antifungal, insecticidal, and antioxidant activities. [1, 9] But till now, no in vivo study has been conducted to examine the effect of Illicium verum directly in the oral cavity of humans, to lookout the anti-inflammatory and astringent property and effect on oral microbial count with its regular usage.

Our study confirmed the effective anti-inflammatory property of Illicium verum by recording gingival status before and after intervention. The study conducted by Deng et al. [5] stated that the Illicium verum aqueous extract has effects of anti-inflammatory and analgesic on mice intestinal smooth muscles. Moreover, a study was conducted by Khan et al. [10] and Oliveira et al. [11] showed the anti-inflammatory activity of Illicium verum as reduced serum levels of IL-1*𝛽* and TNF-*α*.

The present clinical trial investigated the effect of Illicium verum mouth rinse against bacteria present in the microbial ecosystem of the oral cavity, and it was found to be substantial reduction of microbial count. The significant reduction in numbers of oral microorganism also inhibits the growth of various potentially pathogenic microorganisms. This may be due to the presence of supercritical CO_2_ and ethanol extracts in Illicium verum which exert substantial antibacterial activity against Staphylococcus aureus, and this fact was stated by Wang et al. [12] and Benmalek et al. [13] in their respective studies. Singh et al. [14], Chouksey et al. [1], and Peruma et al. [15] conducted a study and proven that the Illicium verum was found to be highly effective against Escherichia coli, Pseudomonas aeruginosa, Bacillus cereus, B. subtilis, and Staphylococcus aureus. Another study by Iauk et al. [16] investigated the in vitro antibacterial activity of methanol extract and decoction of I. verum fruits against anaerobic and facultative aerobic periodontal bacteria and found that E. corrodens and Prevotella spp. have a useful susceptibility to the Illicium verum.

Thejeswar and John et al. [17] in their study reported that both mouthwashes were effective, though chlorhexidine showed better clinical improvement. Herbal mouthwash was found to be comparable to chlorhexidine in reducing bleeding on probing. Thus, herbal mouthwash can be effectively used as an alternative to chlorhexidine and can be prescribed for longer duration without any side effects in children. Herbs, which are powerful healing agents, must be used appropriately. Herbs contain active ingredients that may interact negatively with prescribed medications or other remedies. It is wise, therefore, to consult a health care professional in situations in which you question the appropriateness of the herb or its interaction with other remedies. The use of herbs in dentistry should be based on evidence of effectiveness and safety. The antibacterial activities could be enhanced if active components are purified and adequate dosage determined for proper administration. These results are consistent with other findings by Scherer et al. [18] who demonstrated that herbal mouthwash reduces gingival bleeding over a period of time.

In a study conducted by Amato M et al., the use of alcohol-free chlorhexidine digluconate-based mouthwash with the antipigmentation system, at a concentration of 0.2%, for a period of 2 weeks has been explored, and the authors suggest that it allows for good control of mucobacterial plaque without pigmenting the dental elements [19].

Information on the astringent property of Illicium verum is very scarce. No comprehensive in vivo study has conducted to evaluate the astringent property of Illicium verum intraorally. The current study found that Illicium verum has great astringent property, as bleeding score was decreased significantly.

The limitation of the present study was that subjects were only instructed to follow the regimen but monitoring it was beyond the control of the examiner. Consequently, there might be some irregularity in the way the subjects follow their respective regimen. Another limitation is the short-term follow-up.

## 5. Conclusion

The results of this study suggest the use of the Illicium verum for topical medication in periodontal prophylactics or in the alteration of the microbial ecosystem, as mouthwashes could be a valid aid to obtain a significant reduction of the total microbial population. It is recommended that there is a need for more clinical trials to evaluate the beneficial effects of this plant in human models and synthesis of new drugs from the active ingredients of this plant in future. This in vivo assessment of (anti-inflammatory, astringent property, and effect on oral microbial count) Illicium verum is very promising species for further research focused on development of novel oral care preparations.

## Figures and Tables

**Table 1:** Demographic characteristics of subjects.

Groups	Age (year)		Gender			
	Mean	SD	Male		Female	
			*N*	%	*N*	%
Group A	20.40	1.44	15	60	10	40
Group B	20.56	1.47	14	56	11	44

**Table 2:** Clinical parameters of group A and group B at different stages of study.

Group	Clinical parameters	Baseline 1	First FU	*p* value	Baseline 2	Second FU	*p* value
		Mean ± SD	Mean ± SD		Mean ± SD	Mean ± SD	
A	Gingival index	0.65 ± 0.05	0.48 ± 0.04	0.04^∗^	0.61 ± 0.02	0.60 ± 0.01	0.28
	Papillary bleeding index	0.68 ± 0.05	0.55 ± 0.03	0.02^∗^	0.64 ± 0.03	0.66 ± 0.01	0.09
	Microbial count	0.6 ± 0.06	0.47 ± 0.04	0.01^∗^	0.58 ± 0.04	0.57 ± 0.03	0.26
B	Gingival index	0.63 ± 0.02	0.62 ± 0.03	0.53	0.63 ± 0.05	0.45 ± 0.04	0.04^∗^
	Papillary bleeding index	0.67 ± 0.04	0.69 ± 0.02	0.14	0.70 ± 0.06	0.53 ± 0.03	0.03^∗^
	Microbial count	0.66 ± 0.04	0.67 ± 0.03	0.26	0.68 ± 0.07	0.50 ± 0.05	0.01^∗^

FU: follow-up. ^∗^Statistically significant.

**Table 3:** Intergroup mean comparison for plaque index, gingival index, and microbial count at different stages.

	Group A	Group B	*p* value
Gingival index			
Baseline 1	0.65 ± 0.05	0.63 ± 0.02	0.12
First FU^∗^	0.48 ± 0.04	0.62 ± 0.03	0.01^∗^
Baseline 2	0.61 ± 0.02	0.63 ± 0.05	0.06
Second FU^∗^	0.60 ± 0.01	0.45 ± 0.04	0.01^∗^
Papillary bleeding index			
Baseline 1	0.68 ± 0.05	0.67 ± 0.04	0.43
First FU^∗^	0.55 ± 0.03	0.69 ± 0.02	0.01^∗^
Baseline 2	0.64 ± 0.03	0.70 ± 0.06	0.07
Second FU^∗^	0.66 ± 0.01	0.53 ± 0.03	0.02^∗^
Microbial count			
Baseline 1	0.64 ± 0.06	0.66 ± 0.04	0.17
First FU^∗^	0.47 ± 0.04	0.67 ± 0.03	0.01^∗^
Baseline 2	0.58 ± 0.04	0.68 ± 0.07	0.01^∗^
Second FU^∗^	0.57 ± 0.03	0.50 ± 0.05	0.04^∗^

FU: follow-up. ^∗^Statistically significant.

**Table 4:** Intergroup and intragroup mean difference comparison for plaque index, gingival index, and microbial count.

Mean difference	Group A	Group B	*p* value
	Mean ± SD	Mean ± SD	
Gingival index			
Baseline 1—first FU^∗^	0.17 ± 0.03	0.1 ± 0.02	<0.01^∗^
Baseline 2—second FU^∗^	0.1 ± 0.02	0.18 ± 0.04	<0.01^∗^
*p* value	<0.01^∗^	<0.01^∗^	
Papillary bleeding index			
Baseline 1—first FU^∗^	0.13 ± 0.04	0.2 ± 0.03	<0.01^∗^
Baseline 2—second FU^∗^	0.2 ± 0.02	0.17 ± 0.05	<0.01^∗^
*p* value	<0.01^∗^	<0.01^∗^	
Microbial count			
Baseline 1—first FU^∗^	0.17 ± 0.05	0.1 ± 0.03	<0.01^∗^
Baseline 2—second FU^∗^	0.1 ± 0.03	0.18 ± 0.06	<0.01^∗^
*p* value	<0.01^∗^	<0.01^∗^	

FU: follow-up. ^∗^Statistically significant.

## Data Availability

All research related data can be made readily available on reasonable request.
